# Hsa_circ_0001361 facilitates the progress of lung adenocarcinoma cells via targeting miR-525-5p/VMA21 axis

**DOI:** 10.1186/s12967-021-03045-4

**Published:** 2021-09-10

**Authors:** Hong-Yu Shen, Liu-Xi Shi, Lin Wang, Le-Ping Fang, Wei Xu, Ju-Qing Xu, Bo-Qiang Fan, Wei-Fei Fan

**Affiliations:** 1 Department of Hematology and Oncology, Department of Geriatric Lung Cancer Laboratory, Geriatric Hospital of Nanjing Medical University, Jiangsu Province Geriatric Hospital, No.65 Jiangsu Road, Gulou District, Nanjing, 210000 Jiangsu Province People’s Republic of China; 2 GCP office, The Second Affiliated Hospital of Soochow University, Suzhou, 215000 Jiangsu Province People’s Republic of China; 3 Department of Oncology, The First Affiliated Hospital of Nanjing Medical University, Nanjing, 210000 Jiangsu Province People’s Republic of China

**Keywords:** circ_0001361, LUAD, miR-525-5p, VMA21, Proliferation, Metastasis

## Abstract

**Background:**

Lung adenocarcinoma (LUAD) is a common subtype of lung cancer with high recurrence rate and fatality. Circ_0001361 has been recognized as key regulators in various malignancies, but its roles in LUAD remain ambiguous.

**Methods:**

Circ_0001361, miR-525-5p, and VMA21 levels were assessed by RT-qPCR. The growth and metastasis of LUAD cells were detected by MTT, colony formation, wound scratch, and transwell assays, respectively. The interaction between circ_0001361/VMA21 and miR-525-5p was detected by dual luciferase, RNA immunoprecipitation, and RNA pull-down assays. VMA21 protein level was detected by Western blotting. Nude mouse xenograft model was established to determine the role of circ_0001361 in tumor growth in vivo.

**Results:**

Circ_0001361 was up-regulated, while miR-525-5p was down-regulated in LUAD tissues and cells. Functional experiments demonstrated that circ_0001361 drove LUAD cell growth and metastasis. Mechanistically, circ_0001361 functioned as a sponge of miR-525-5p to up-regulate downstream target VMA21 level. MiR-525-5p/VMA21 axis was involved in circ_0001361-mediated malignant phenotypes of LUAD cells. Finally, inhibition of circ_0001361 restrained in vivo xenograft tumor growth via regulating miR-525-5p/VMA21 axis.

**Conclusion:**

Our findings elucidate that circ_0001361 facilitates the tumorigenesis and development of LUAD through miR-525-5p/VMA21 axis, providing evidence for circ_0001361 as a potential prognosis biomarker and therapeutic target for clinical treatment of LUAD.

**Supplementary Information:**

The online version contains supplementary material available at 10.1186/s12967-021-03045-4.

## Introduction

Lung adenocarcinoma (LUAD), assigned to non-small cell lung cancer, is the most subtype of lung cancer, accounting for 40% of all lung cancer patients[1]. LUAD cells originate from respiratory tract epithelial, type II alveolar cells[2]. It is well established that LUAD occurs in people of all ages, and smoking is a risk factor of developing lung cancer (and LUAD), even if LUAD can occur in never smoker patients[3]. Due to the lack of noticeable symptoms, most LUAD patients were diagnosed in advanced stages with metastasis[4]. Despite the progress of clinical techniques, the 5-year survival rate for LUAD patients is 15–17%[5]. Therefore, uncovering the pathogenesis mechanisms and identifying potential targets are urgently needed to prevent the progression of LUAD.

Circular RNAs (circRNAs), unlike conventional linear RNAs, are a new type of non-coding RNAs that shape a closed loop structure[6]. CircRNAs are abundantly and conservatively expressed in eukaryotic cells, which possess multiple pathophysiological functions[7]. Growing evidence has shown that circRNAs are implicated in the occurrence and development of tumors[8]. It has been discovered that circRNAs are dysregulated in multiform types of cancers, and may be served as biomarkers and therapeutic targets for cancer[9]. For example, Fu et al. demonstrated that hsa_circRNA_012515 level was up-regulated in non-small cell lung cancer tissues, which indicated the poor prognosis of the patients[10]. Circ-SMAD7 has been revealed to be down-regulated in colorectal cancer tissues, and overexpression of circ-SMAD7 repressed the migration and invasion via inhibiting epithelial-to-mesenchymal transition in colorectal cancer cells[11]. Sponging miRNAs, which results in inhibition of target miRNA expression and subsequent enhancing levels of miRNA-targeted genes has been considered to be the main regulatory mechanism of circRNAs[12]. Increasing studies have revealed that circRNAs play crucial roles in affecting the malignant phenotypes of LUAD, including growth and metastasis[13, 14]. A recent research by Feng et al. suggested that hsa-circ-0000211 drove the migration and invasion of LUAD cells via enhancing HIF1-α expression by sequestering miR-622[15]. Circ-TSPAN4 facilitated the metastasis of LUAD via serving as a sponger of miR-665 to increase ZEB1 expression[16]. Recently, up-regulation of hsa_circ_0001361 has been found in bladder cancer, which contributed to the enhanced metastatic potential and might be served as a target for bladder cancer therapy [17]. In addition, circ_0001361 was reported to be up-regulated in hepatocellular carcinoma[18]. Bian et al. revealed that FNDC3B, the host gene of circ_0001361, could contribute to LUAD progression via regulating epithelial-mesenchymal transition[19]. However, the regulatory functions and underlying mechanisms of circ_0001361 in LUAD have not been clarified.

Vacuolar ATPase assembly factor VMA21 (VMA21) is a key assembly chaperone of vacuolar ATPase. The participation of VMA21 in cancer process has been reported. For instance, VMA21 down-regulation delayed LINC00665/miR-224-5p axis-mediated melanoma progression[20]. Xue et al. revealed that VMA21 was up-regulated in LUAD cells as compared with human bronchus epithelial cells, and ZFPM2-AS1/miR-18b-5p targeting VMA21 facilitated the growth of LUAD cells[21]. In addition, StarBase database also indicated that VMA21 expression was increased in LUAD samples. ceRNA has been accepted as one of pivotal mechanisms of circRNAs to regulate tumor progression. CircRNA can competitively bind to miRNA to enhance mRNA expression, which takes part in tumor development[22]. Our preliminary experiment found that circ_0001361 level was elevated in LUAD samples. Interestingly, both circ_0001361 and VMA21 were predicted to bind with miR-525-5p that was down-regulated in LUAD. Therefore, we speculated that circ_0001361 might sponge miR-525-5p to favor the progression of LUAD via regulating VMA21 expression, and VMA21 was selected for the study of the function of circ_0001361.

Hence, we sought to elucidate the expression and function of circ_0001361 in LUAD. In the present study, the abnormal up-regulation of circ_0001361 was firstly found in LUAD tissues and cell lines. In addition, we discovered that circ_0001361 sequestered miR-525-5p to enhance vacuolar ATPase assembly factor VMA21 (VMA21) expression, which participated in the proliferation, migration and invasion of LUAD cells. Our findings uncover the novel pathogenesis of LUAD, and suggest that circ_0001361 may be considered as a potential therapeutic target for LUAD.

## Materials and methods

### Clinical samples

The expression of circ_0001361 was determined in 32 pairs of LUAD tissues and matched non-cancerous lung tissues collected from patients with informed consents from Geriatric Hospital of Nanjing Medical University between January 2017 and January 2019. Chemotherapy or radiotherapy was not given to these patients before surgery. All experiments were permitted by the Ethics Committee of Geriatric Hospital of Nanjing Medical University.

### Cell lines and cell culture

Normal human bronchial epithelial (HBE) cells, A549, H1975, PC9, and Calu-3 cells were obtained from the cell bank of Cancer Research Institute of Central South University (Hunan, China). All cells received routine mycoplasma test were cultured in RPMI-1640 supplemented with 10% fetal bovine serum at 37 °C containing 5% CO_2_.

### Cell transfection

The expression plasmid of VMA21 was constructed by cloning cDNA sequences into pcDNA3.1 vector. The circ_0001361 overexpression plasmid was obtained from Geenseed Biotech (Guangzhou, China) [17]. pGPU6-shRNA targeted circ_0001361 (sh-circ_0001361), pGPU6-shRNA negative control (sh-NC), sh-VMA21, miR-525-5p mimics, mimics negative control (mimic NC), miR-525-5p inhibitor, and inhibitor NC were synthesized by Shanghai GenePharma Co., Ltd. Transfection of the above plasmids and oligonucleotides was carried out using Lipofectamine 3000 (Thermo Fisher, Waltham, MA, USA). Stable transfected cells were selected using puromycin after the transient transfection and then were utilized in subsequent experiments.

### Preparation of nuclear and cytoplasmic RNA

The nuclear and cytoplasmic RNA was extracted using a PARIS Kit (Thermo Fisher). In brief, cells were lysed and centrifuged to obtain the nuclear and cytoplasmic fractions, followed by adding with 2× Lysis/Binding Solution and 100% ethanol. Then the mixture was filtered and eluted before subsequent detection.

### RT-qPCR

TRIzol reagent (Thermo Fisher) was adopted for the isolation of total RNA from tissues and cells. Subsequently, cDNA was generated using PrimeScript RT Master Mix (Takara, Osaka, Japan) with random oligo for circRNA and mRNA, or using miRcute Plus miRNA First-Strand cDNA Kit (TIANGEN, Beijing, China) for miRNA. qRT‐PCR reactions were conducted using SYBR Green Realtime PCR Master Mix (SinoBio, Shanghai, China) for circRNA and mRNA, or miRcute miRNA qPCR Detection Kit (TIANGEN) for miRNA. PCR primers were as follows: circ_0001361: 5′‐GAGATGCAGCTCAGCAGGTTA‐3′ (forward) and 5′‐AATGGTGGCAGTTCCAGAGG‐3′ (reverse); VMA21: 5′‐AGACGCTCCTGTTCTTCACA‐3′ (forward) and 5′‐CATACACAAAGAGGGCCAGC‐3′ (reverse); 18S rRNA: 5′‐CTTGGTCATTTAGAGGAAGTAA‐3′ (forward) and 5′‐GCTGCGTTCTTCATCGATGC‐3′ (reverse); miR-525-5p: 5′‐GGCTCCAGAGGGATGCA‐3′ (forward) and 5′‐GTCGTATCCAGTGCAGGGTCCGAGGTATTCGCACTGGATACGACAGAAAG‐3′ (reverse); U6: 5′‐GCTTCGGCAGCACATATACTAAAAT‐3′ (forward) and 5′‐CGCTTCACGAATTTGCGTGTCAT‐3′. (reverse). Circ_0001361 and VMA21 expression levels were normalized to 18S rRNA, and miR-525-5p expression was normalized to U6. 2^−ΔΔCT^ method was used for the analysis of data.

### Cell proliferation assay

To assess the proliferation of A549 and PC9 cells, MTT assay was adopted. After incubation for the indicated time points, A549 and PC9 cells in 96-well plates were added with MTT (5 mg/mL, Amresco, Solon, OH, USA) and maintained at 37 °C for 4 h, followed by dissolving in 100 μL of dimethylsulfoxide (DMSO). The absorbance at 490 nm was detected on a microplate reader (Thermo Fisher).

### Colony formation assay

A549 and PC9 cells subjected to various transfections were seeded in 6-well plates (1000 cells per well) and cultured for 14 days. Then the cells were immersed in 0.1% crystal violet (Beyotime, Haimen, China) for staining and imaged under a microscope (Olympus, Tokyo, Japan).

### Transwell invasion assay

The invasion ability of A549 and PC9 cells was detected by Transwell chambers (8 μm, Corning, Tewksbury, MA, USA) with Matrigel matrix (Corning). Briefly, the cells resuspended in serum-free medium were placed into the upper chambers. The lower chambers were added with culture medium containing 10% fetal bovine serum. After incubating in a 37 °C incubator for 24 h, the invaded cells were stained with 0.1% crystal violet, photographed and counted.

### Wound scratch assay

A549 and PC9 cells were seeded in 24-well plates. After reaching full confluence, a scratch was made using a 200-µl pipette tip. The floating cells were removed by washing with phosphate-buffered saline. After incubation in serum-free medium for 24 h, the migrated cells were photographed and quantitatively analyzed.

### Dual luciferase assay

The predicted interaction between miR-525-5p and circ_0001361/VMA21 was verified by dual luciferase assay. The sequences of circ_0001361/VMA21 containing the predicted or mutant binding sites for miR-525-5p was inserted into pmirGLO vector (Promega, Madison, WI, USA). The constructed plasmids circ_0001361/VMA21-wild type (WT) containing miR-525-5p binding sites or circ_0001361/VMA21-mutant (MUT) containing mutant miR-525-5p binding sites together with miR-525-5p mimics or mimic NC were co-transfected into A549 cells. The luciferase activity in cells after the transfection for 24 h was evaluated using Dual-Lucy Assay Kit (Solarbio, Beijing, China).

### RNA immunoprecipitation (RIP) assay

The combination between circ_0001361 and miR-525-5p was determined by RIP assay using an Imprint RNA Immunoprecipitation kit (Merck Millipore, Billika, MA, USA). Briefly, the collected cells were suspended in lysis buffer. After centrifuging at 12,000 g for 30 min, Ago-2 antibody or IgG antibody pre-bound to magnetic beads was added to cells. After incubation over night at 4 °C, RNA was eluted and RT-qPCR was carried out to assess the expression of circ_0001361.

### RNA pull-down assay

For RNA pull-down assay, biotinylated WT miR-525-5p (bio-miR-525-5p-WT) or mutant miR-525-5p (bio-miR-525-5p-MUT) was transcribed into the cells. After the transfection for 48 h, the cells were collected and suspended in IP Cell lysis Buffer (Sangon, Shanghai, China) containing protease inhibitor cocktail for 10 min on ice. Subsequently, biotin-coupled RNA complex was purified after the incubation with streptavidin-coated magnetic beads (Thermo Fisher) at 4 °C for 3 h. The abundance of circ_0001361 was determined by RT-qPCR.

### Western blotting

The tissues and cells were lysed in RIPA buffer (Solarbio) supplemented with protease inhibitor cocktail (Solarbio). After protein quantification using BCA protein assay kit (Solarbio), the lysates with equal amount protein were subjected to SDS-PAGE and transferred to polyvinylidene difluoride membranes (Roche, Basel, Switzerland). The membranes were immersed in 5% skim milk for 1 h, and then probed with the antibodies against VMA21 (1:500, 21921-1-AP, Proteintech, Wuhan, China) and GAPDH (1:5000, 10494-1-AP, Proteintech) overnight at 4 °C. Then HRP-conjugated Goat Anti-Rat IgG (1:2000, SA00001-15, Proteintech) was applied to the membranes. The membranes were visualized using ECL Western Blotting Substrate (Solarbio).

### Mouse xenograft model

Male BALB/c nude mice were purchased from Hunan Slac Jingda Laboratory Animal Co., Ltd. (Hunan, China). A549 and PC9 cells infected with lentivirus expressing sh- circ_0001361 or sh-NC (Genechem, Shanghai, China) were subcutaneously inoculated into the mice. The tumor size was measured every 4 days and the volume of tumors was calculated as following: volume = (length × width^2^)/2. All mice were euthanized at 5 weeks after the inoculation. The tumors were resected, weighed, and the expression levels of circ_0001361, miR-525-5p, and VMA21 were assessed by RT-qPCR and Western blotting. All animal experiments were approved by the ethics Geriatric Hospital of Nanjing Medical University.

### Statistical analysis

Data from at least three independent experiments are shown as mean ± standard deviation (SD). Student’s *t* test or one-way analysis of variance followed by Bonferroni’s multiple comparison test was adopted for statistical analysis using GraphPad Prism 8 software. Pearson correlation coefficient was used to analyze the relationship among circ_0001361, miR-525-5p and VMA21. A *P* value less than 0.05 was set as statistically significant.

## Results

### Expression and distribution of circ_0001361 in LUAD tissues and cells

**Fig. 1:**
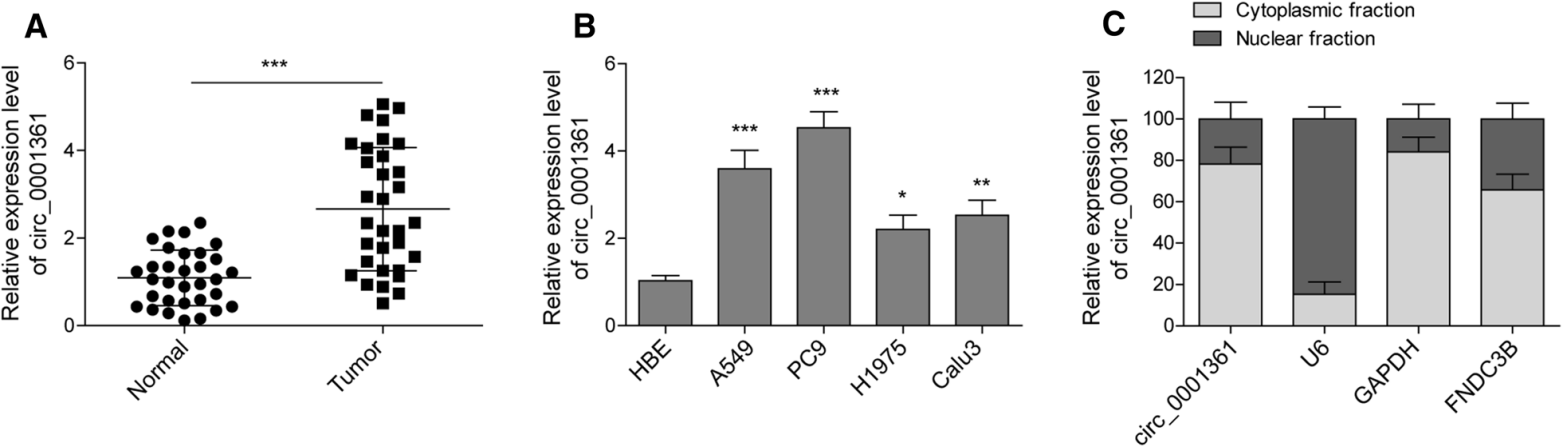
Expression of circ_0001361 in LUAD tissues and cells. **A** The expression of circ_0001361 in LUAD tissues and corresponding adjacent normal lung tissues was detected by RT-qPCR. **B** The expression of circ_0001361 in normal human bronchial epithelial cells (HBE), and LUAD cell lines (A549, H1975, Calu-3, and PC9) was determined by RT-qPCR. **C** The expression of circ_0001361 and its host gene FNDC3B in the nucleus and cytoplasm of A549 and PC9 cells was evaluated by RT-qPCR. All data are expressed as the means ± standard deviation from three independent experiments. *P < 0.05; **P < 0.01; ***P < 0.001 versus the indicated group

The expression level of circ_0001361 in paired LUAD tissues and adjacent non-cancerous lung tissues was evaluated by RT-qPCR. As shown in Fig. 1A, a significant higher level of circ_0001361 was found in LUAD tissues as compared with that in adjacent non-cancerous lung tissues. Moreover, circ_0001361 level was remarkably enhanced in LUAD cells, particularly in A549 and PC9 cells, compared with that in HBE cells (Fig. 1B). We further investigated the cell distribution of circ_0001361 in LUAD cells. As presented in Fig. 1C, circ_0001361 and its host gene FNDC3B were highly expressed in the cytoplasm of LUAD cells. These results indicated that circ_0001361, mainly locating in the cytoplasm, was highly expressed in LUAD tissues and cells.

### Down-regulation of circ_0001361 suppresses the growth, migration and invasion of LUAD cells

**Fig. 2:**
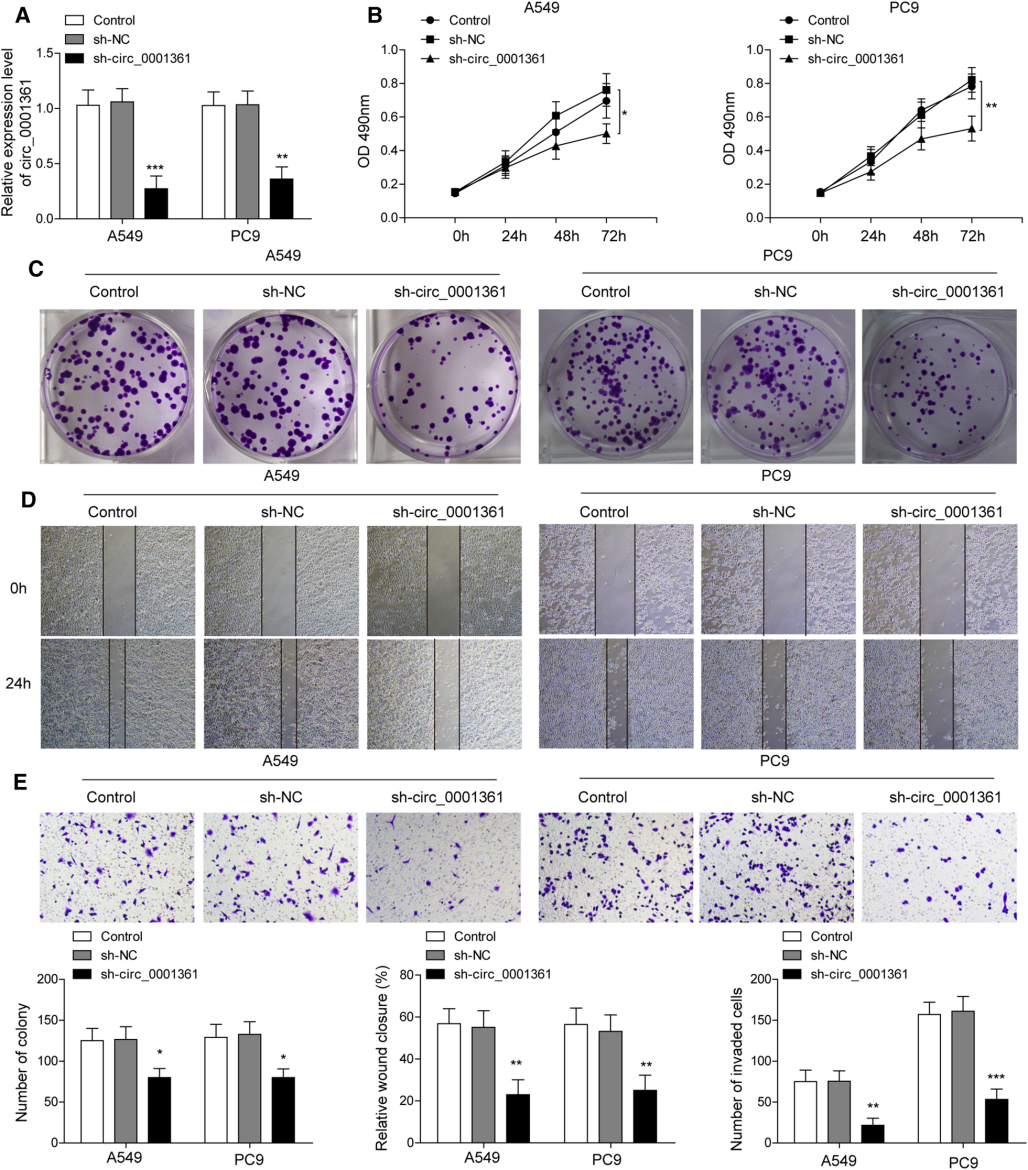
The biological function of circ_0001361 in LUAD cells. **A** The interference efficiency was determined by RT-qPCR. **B** MTT assay for determining the proliferation of A549 and PC9 cells. **C** Colony formation assay for evaluating the clonogenic ability of A549 and PC9 cells. **D** The migration of A549 and PC9 cells was assessed by wound scratch assay. **E** The invasive ability of A549 and PC9 cells was detected by Transwell assay. All data are expressed as the means ± standard deviation from three independent experiments. *P < 0.05; **P < 0.01; ***P < 0.001 versus the sh-NC group

Next, we elucidated the biological functions of circ_0001361 in LUAD cells through stably transfecting sh-circ_0001361 into A549 and PC9 cells to reduce circ_0001361 expression. As assessed by RT-qPCR, sh-circ_0001361 transfection led to an obvious decrease of circ_0001361 expression in A549 and PC9 cells (Fig. 2A). Furthermore, MTT assay revealed that the proliferation of A549 and PC9 cells was restrained by knockdown of circ_0001361 (Fig. 2B). Consistently, the clonogenic ability of LUAD cells was suppressed by silencing of circ_0001361 (Fig. 2C). In addition, wound scratch assay indicated that depletion of circ_0001361 resulted in decreased migration ability of A549 and PC9 cells (Fig. 2D). As presented in Fig. 2E, transwell assay further demonstrated that down-regulation of circ_0001361 inhibited the invasion of LUAD cells. Taken together, these observations suggested that circ_0001361 silencing inhibited the proliferation, migration, and invasion of LUAD cells.

### Circ_0001361 serves as a sponge of miR-525-5p

**Fig. 3:**
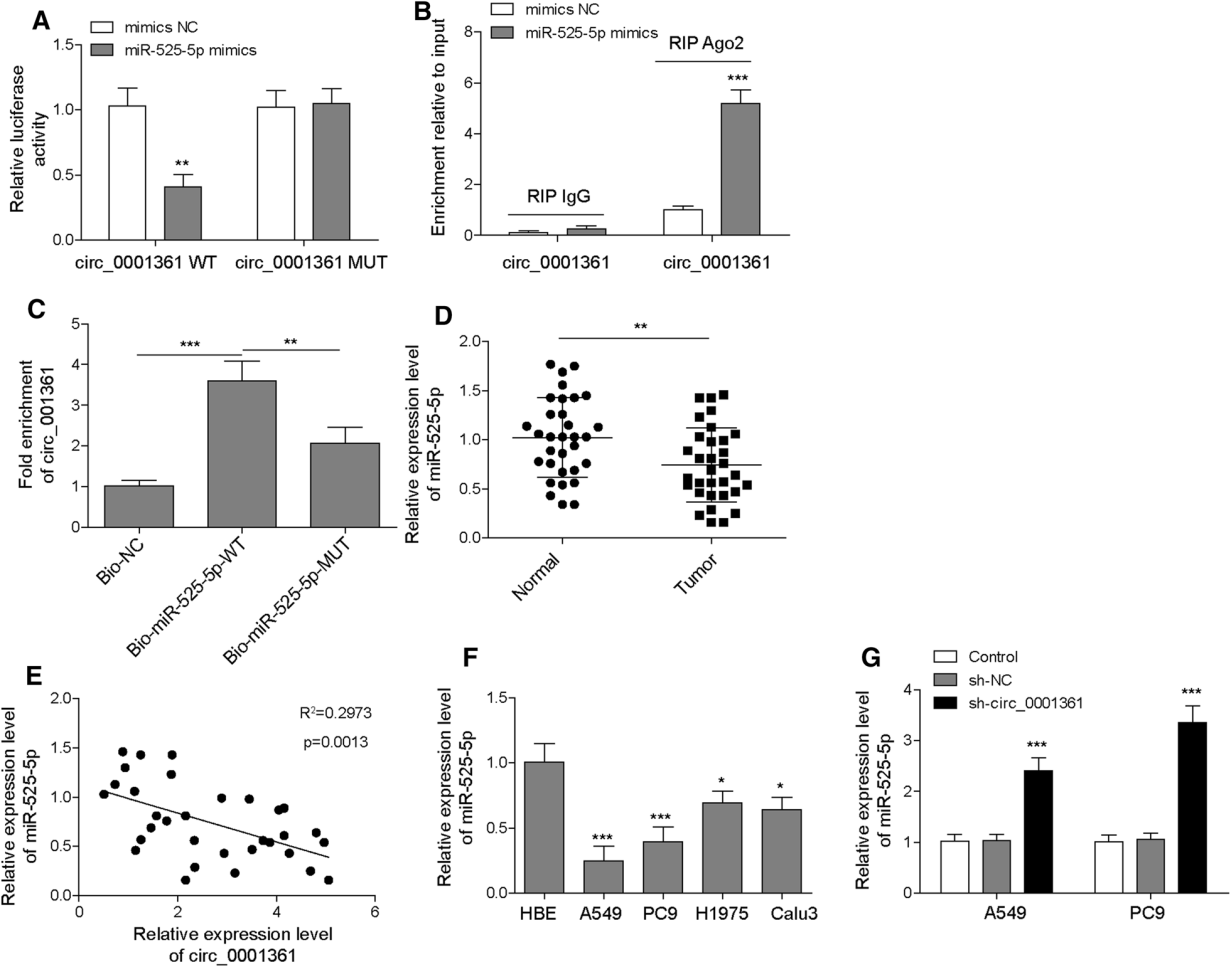
Circ_0001361 sponges miR-525-5p in LUAD cells. **A** The relationship between miR-525-5p and circ_0001361 was verified by dual luciferase assay. **B** RIP assay assessed the interaction between circ_0001361 and miR-525-5p in A549 cells. **C** RNA pull-down assay was performed in A549 cells using biotinylated miR-525-5p probe, and subsequent RT-qPCR for determining the enrichment of circ_0001361. **D** RT-qPCR for the expression of miR-525-5p in LUAD and normal adjacent tissues. **E** The correlation between circ_0001361 and miR-525-5p was analyzed. **F** The level of miR-525-5p in normal human bronchial epithelial cells and LUAD cells was determined by RT-qPCR. **G** The expression of miR-525-5p in A549 and PC9 cells after transfection with sh-circ_0001361 was evaluated by RT-qPCR. All data are expressed as the means ± standard deviation from three independent experiments. *P < 0.05; **P < 0.01; ***P < 0.001 versus the indicated group

It has been reported that circ_0001361 could function as a miRNA sponge to affect the progression of bladder cancer[17]. By bioinformatics analysis, we also found that miR-525-5p possessed complementary binding sites with circ_0001361 (Additional file 1: Fig. S1A). By overlapping the bioinformatics analysis results, only two miRNAs (miR-525-5p and miR-520a-5p) can bind to both circ_0001361 and VMA21. In addition, we found that knockdown of circ_0001361 resulted in a more significant increase in miR-525-5p expression than miR-520a-5p (Additional file 1: Fig. S1C). Thus, miR-525-5p was selected in this study. As confirmed by luciferase reporter assay, miR-525-5p significantly reduced luciferase activity in circ_0001361-WT group, whereas it did not change that in circ_0001361-MUT group, confirming the interaction between circ_0001361 and miR-525-5p (Fig. 3A). Additionally, RIP assay showed that the enrichment of circ_0001361 by Ago2 was evidently increased after the transfection with miR-525-5p mimics (Fig. 3B). Moreover, RNA pull-down assay was performed to further verify the binding between miR-525-5p and circ_0001361. As illustrated in Fig. 3C, circ_0001361 was significantly enriched in Bio-miR-525-5p-WT group, compared with Bio-miR-525-5p-MUT group. Subsequently, a distinct decreased expression of miR-525-5p was observed in the LUAD tissues (Fig. 3D). There was a negative correlation between circ_0001361 and miR-525-5p expression in LUAD samples (Fig. 3E). Consistently, a down-regulation of miR-525-5p was observed in a series of LUAD cells (Fig. 3F). Furthermore, miR-525-5p level was strikingly up-regulated by silencing of circ_0001361 in A549 and PC9 cells (Fig. 3G). Therefore, these results indicated that circ_0001361 could directly bind to miR-525-5p in LUAD cells.

### Circ_0001361 facilitates the malignant phenotypes of LUAD cells via sponging miR-525-5p to increase VMA21 expression

**Fig. 4:**
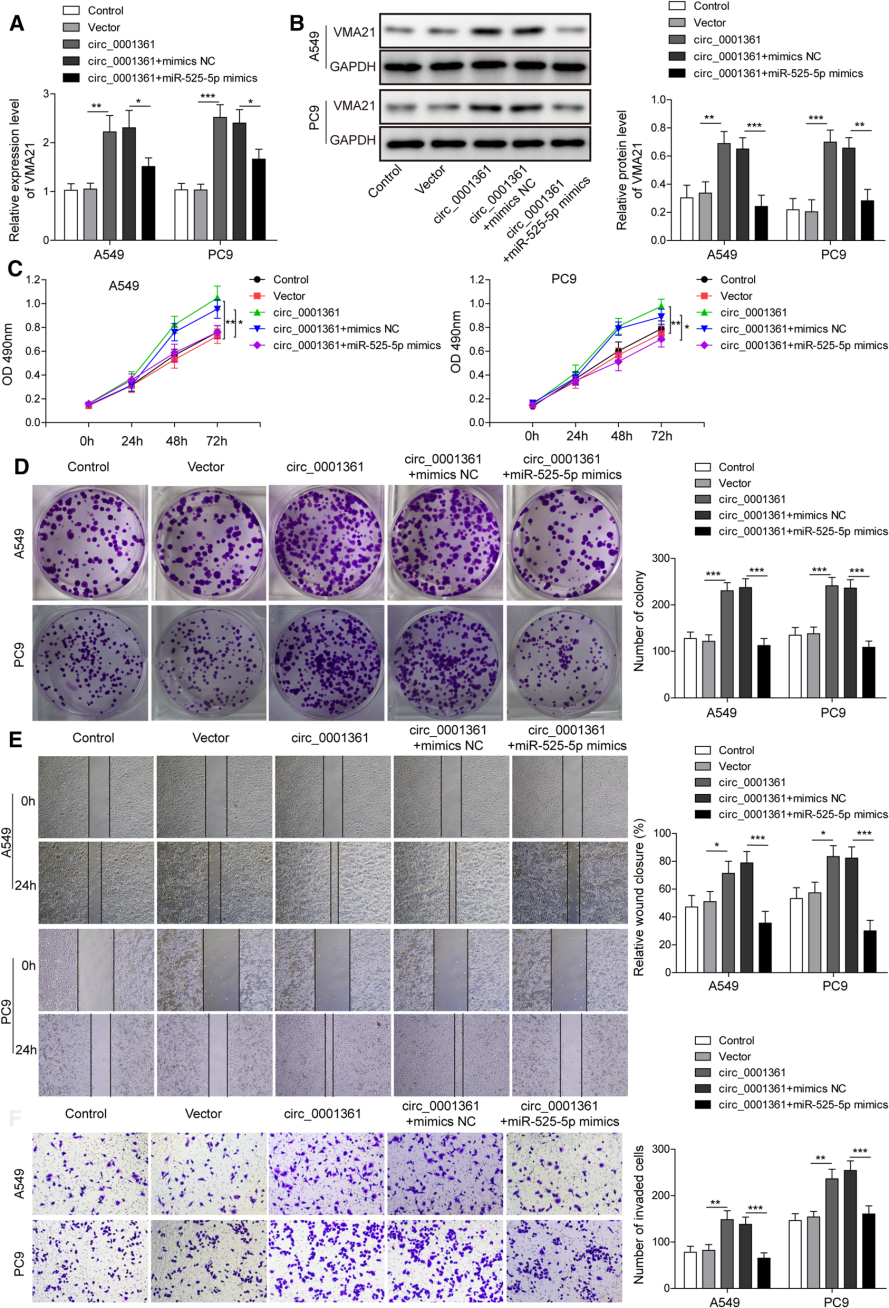
Circ_0001361 facilitates the progression of LUAD cells via sponging miR-525-5p to increase VMA21 expression. RT-qPCR (**A**) and Western blotting (**B**) for the expression of VMA21 in A549 and PC9 cells from different groups. The proliferation of A549 and PC9 cells was determined by MTT assay (**C**) and colony formation assay (**D**). **E** Wound scratch assay for the migration of A549 and PC9 cells with various treatments. **F** The invasive ability of A549 and PC9 cells was detected by Transwell assay. All data are expressed as the means ± standard deviation from three independent experiments. *P < 0.05; **P < 0.01; ***P < 0.001 versus the indicated group

Next, the underlying regulatory mechanisms of circ_0001361 in the progression of LUAD were investigated. As shown in Fig. 4A and B, the mRNA and protein expression of VMA21 in A549 and PC9 cells was raised by overexpression of circ_0001361, while miR-525-5p mimics reversed this effect. In addition, overexpression of circ_0001361 promoted the proliferation and colony formation of A549 and PC9 cells, which were counteracted by miR-525-5p mimics (Fig. 4C and D). Additionally, exogenous expression of miR-525-5p also suppressed the enhanced migration and invasion abilities in circ_0001361-overexpressed A549 and PC9 cells (Fig. 4E and F). Collectively, these results proved that circ_0001361 promoted the progression of LUAD cells via regulating miR-525-5p/VMA21 axis.

### MiR-525-5p represses the growth and metastasis of LUAD cells via targeting VMA21

**Fig. 5:**
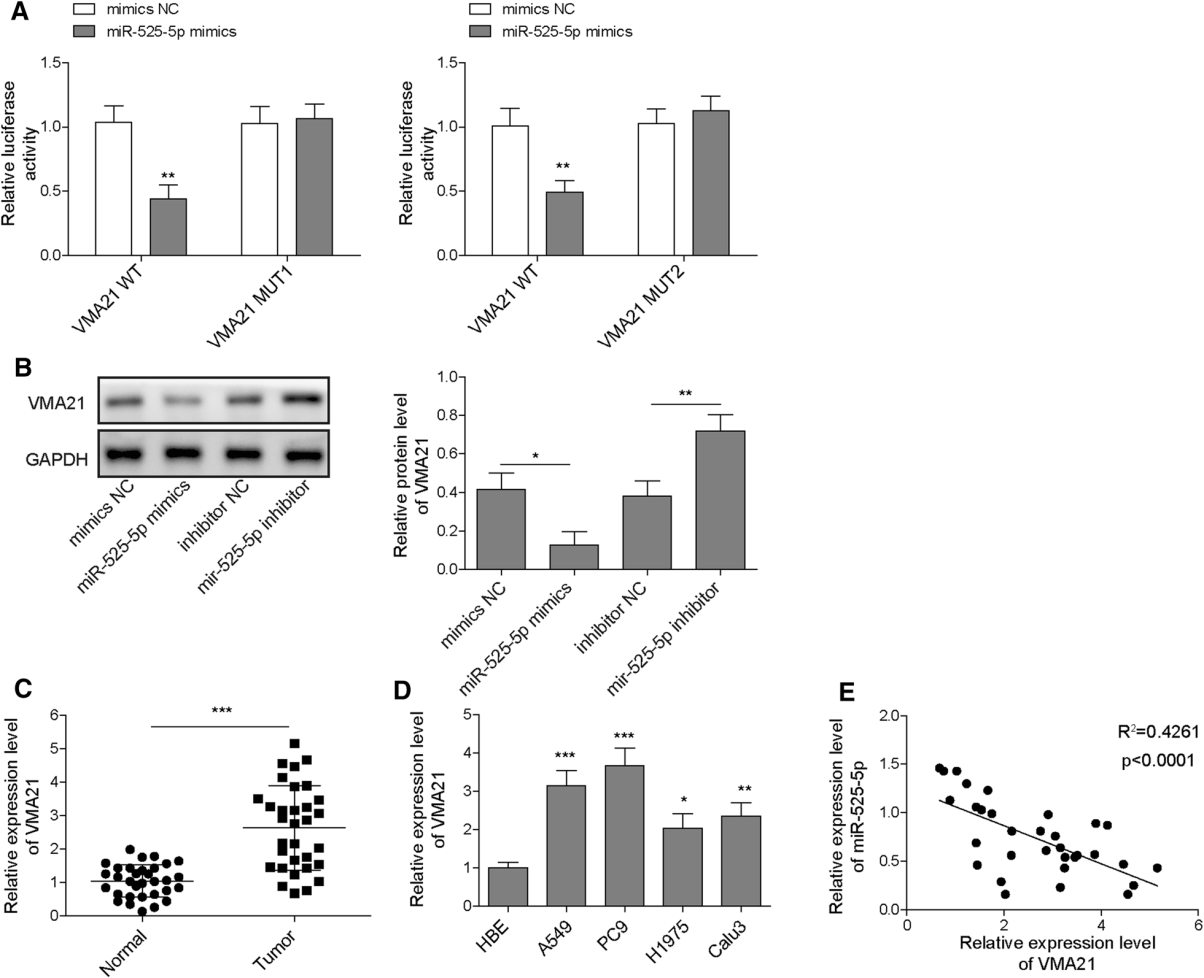
MiR-525-5p targets VMA21 and inhibits VMA21 expression in LUAD cells. **A** The interaction between miR-525-5p and VMA21 was assessed by dual luciferase assay. **B** Western blotting for the protein level of VMA21 in A549 cells transfected with miR-525-5p inhibitor or mimics. **C** RT-qPCR for determining VMA21 expression in LUAD and normal adjacent tissues. **D** The VMA21 level in multiple LUAD cells and normal human bronchial epithelial cells was detected by RT-qPCR. **E** The correlation between VMA21 and miR-525-5p was analyzed. All data are expressed as the means ± standard deviation from three independent experiments. *P < 0.05; **P < 0.01; ***P < 0.001 versus the indicated group

**Fig. 6:**
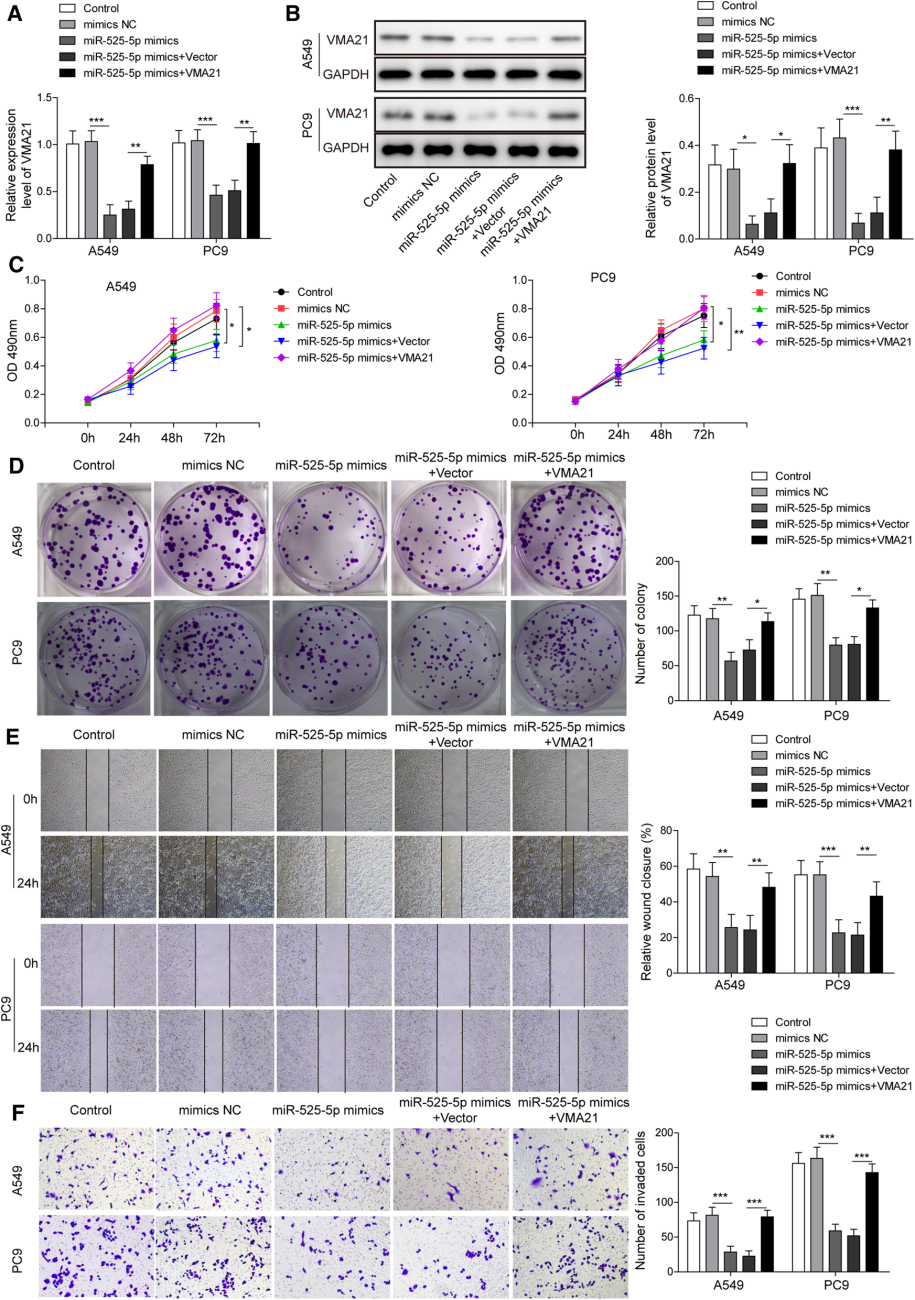
Overexpression of VMA21 reverses miR-525-5p-mediated anti-tumor effects on LUAD. A549 and PC9 cells were transfected with mimic NC or miR-525-5p mimics with or without vector or VMA21 plasmid. RT-qPCR (**A**) and Western blotting (**B**) for evaluating the levels of miR-525-5p and VMA21 in A549 and PC9 cells. **C** The growth of A549 and PC9 cells receiving multiple transfections was detected by MTT assay. **D** Colony formation assay for determining the oncogenicity of A549 and PC9 cells. **E** The migration ability was assessed by wound scratch assay. **F** Transwell assay for evaluating the invasion of A549 and PC9 cells. All data are expressed as the means ± standard deviation from three independent experiments. *P < 0.05; **P < 0.01; ***P < 0.001 versus the indicated group

TargetScan database predicted that there were two binding sites between miR-525-5p and VMA21 (Additional file 1: Fig. S1B). Further dual luciferase assay verified that miR-525-5p mimics resulted in decreased luciferase activity in VMA21-WT group, whereas this effect was eliminated in VMA21-MUT group (Fig. 5A). Moreover, Western blotting showed that the protein level of VMA21 in A549 cells was down-regulated by miR-525-5p mimics, but up-regulated by miR-525-5p inhibitor (Fig. 5B). Thus, miR-525-5p could regulate VMA21 expression in A549 cells via direct binding to the 3’ untranslated region. In addition, RT-qPCR results showed that VMA21 expression was increased in LUAD tissues and cells (Fig. 5C and D). In addition, StarBase database also indicated that VMA21 expression was increased in LUAD samples (Additional file 1: Fig. S1D). As presented in Fig. 5E, VMA21 expression negatively correlated with miR-525-5p expression in LUAD samples. However, VMA21 transcript was positively correlated with circ_0001361 (Additional file 1: Fig. S1E). To evaluate whether miR-525-5p could affect the malignant phenotypes of LUAD cells via targeting VMA21, A549 and PC9 cells were transfected with miR-525-5p mimics with or without VMA21 overexpression plasmid. As shown in Fig. 6A and B, overexpression of miR-525-5p led to reduced mRNA and protein expression of VMA21 in A549 and PC9 cells, which could be restored by transfecting VMA21 overexpression plasmid. As assessed by MTT and colony formation assays, the growth of A549 and PC9 cells was remarkably repressed by miR-525-5p mimics, whereas overexpression of VMA21 reversed the growth inhibition induced by miR-525-5p mimics (Fig. 6C and D). Besides, the results of wound scratch assay described that miR-525-5p mimics-induced decrease in migration ability was restored in VMA21-overexpressed A549 and PC9 cells (Fig. 6E). Similarly, A549 and PC9 cells transfected with miR-525-5p mimics displayed lower invasion ability, which could be counteracted by overexpression of VMA21 (Fig. 6F). The above findings revealed that miR-525-5p inhibited the progression of LUAD cells via targeting VMA21.

### Circ_0001361 reversed VMA21 knockdown-mediated inhibition in growth and metastasis of LUAD cells

**Fig. 7:**
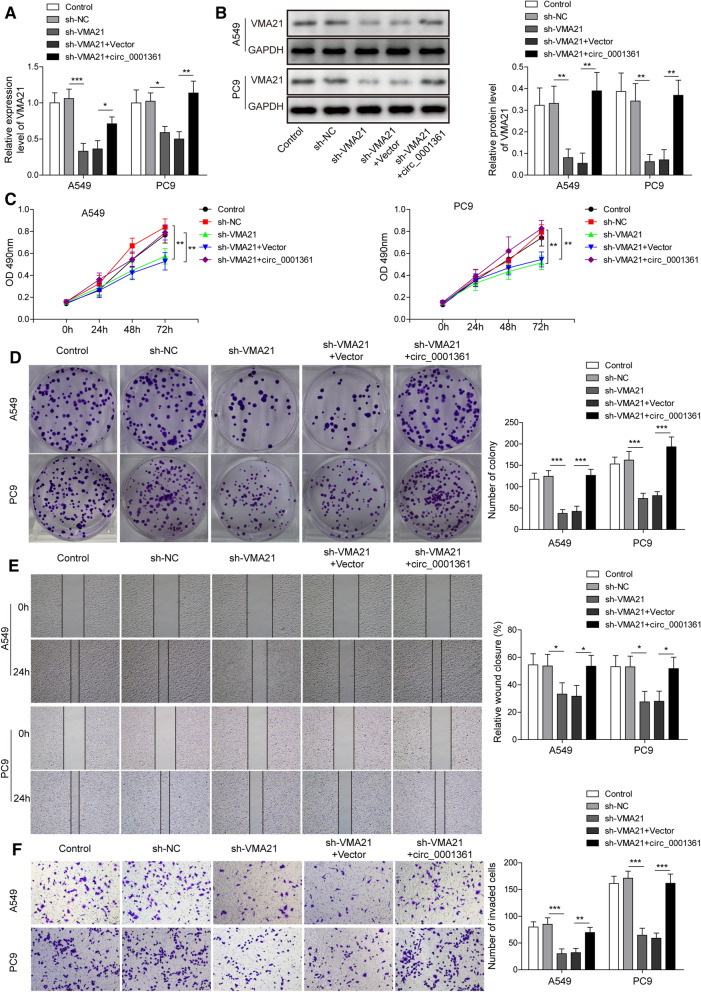
Circ_0001361/VMA21 axis affects the growth and metastasis of LUAD cells. The mRNA and protein expression of VMA21 in A549 and PC9 cells was evaluated by RT-qPCR (**A**) and Western blotting (**B**). MTT assay (**C**) and colony formation assay (**D**) were carried out to investigate the growth of LUAD cells. **E** The migration was determined by wound scratch assay. **F** Transwell assay for assessing the invasion of A549 and PC9 cells. All data are expressed as the means ± standard deviation from three independent experiments. *P < 0.05; **P < 0.01; ***P < 0.001 versus the indicated group

To assess the involvement of circ_0001361/VMA21 axis in the malignant progression of LUAD cells, A549 and PC9 cells were transfected with sh-VMA21 together with or without circ_0001361 plasmid. As illustrated in Fig. 7A and B, sh-VMA21-induced decrease in mRNA and protein levels of VMA21 was partly counteracted by circ_0001361 overexpression. The growth of LUAD cells was repressed by VMA21 knockdown, however, this change could be reversed in circ_0001361-overexpressed cells (Fig. 7C and D). Besides, depletion of VMA21 reduced the migratory and invasive abilities of LUAD cells, which was abolished by circ_0001361 overexpression (Fig. 7E and F). Collectively, circ_0001361/VMA21 axis conferred the growth and metastasis abilities of LUAD cells.

### Circ_0001361 affects xenograft tumor growth via regulating miR-525-5p/VMA21 axis

**Fig. 8:**
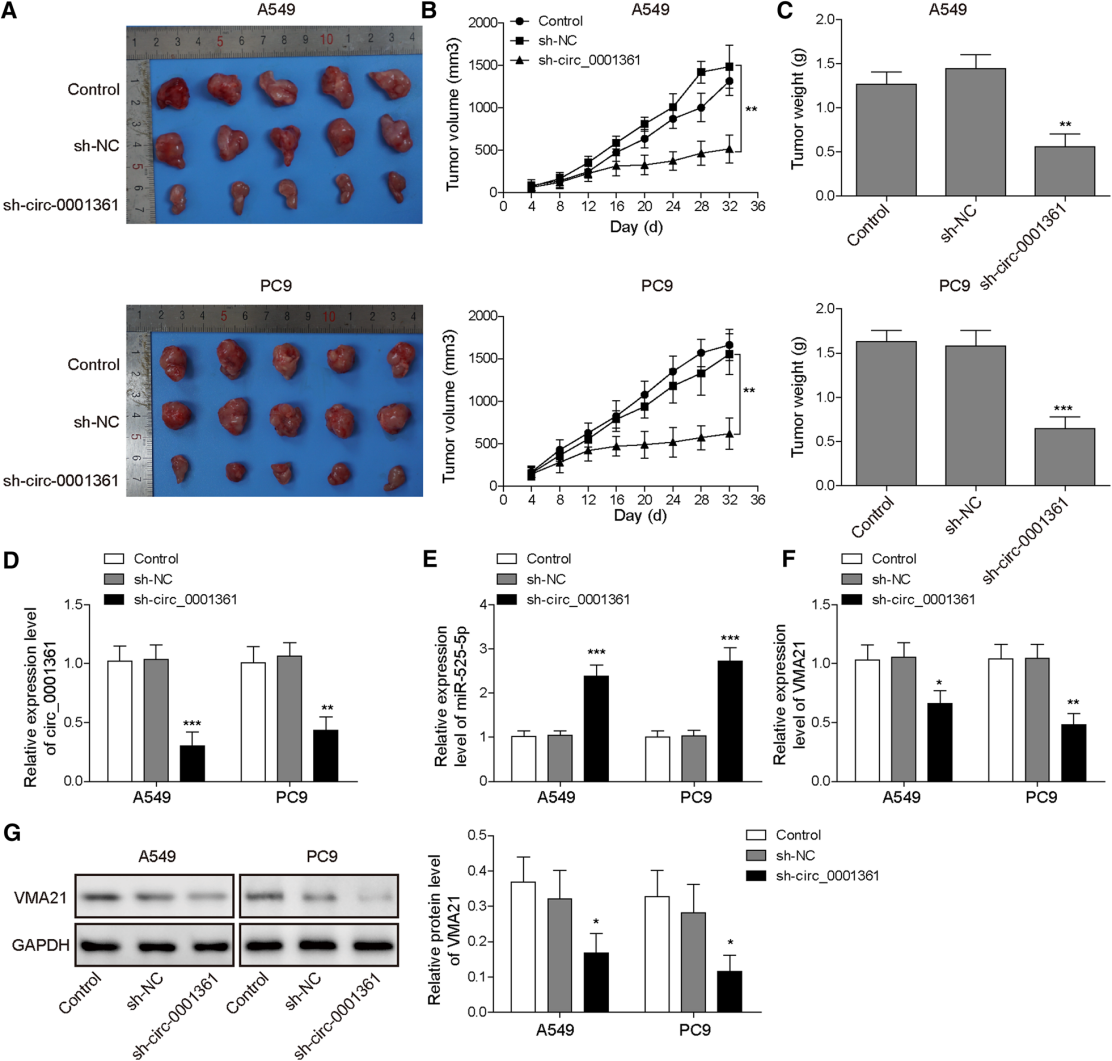
Circ_0001361 silencing restrains in vivo xenograft tumor growth via regulating miR-525-5p/VMA21 axis. **A** Photographs illustrated tumors in xenografts. **B** Tumor volume at the indicated time points was shown. **C** Tumor weight from each group was presented. The expression of circ_0001361 (**D**), miR-525-5p (**E**), and VMA21 (**F**) in tumor tissues was detected by RT-qPCR. **G** Western blotting for VMA21 protein level in tumor tissues. All data are expressed as the means ± standard deviation from three independent experiments. *P < 0.05; **P < 0.01; ***P < 0.001 versus the sh-NC group

To further verify the function of circ_0001361 in xenograft tumor growth in vivo, A549 and PC9 cells infected with lentivirus expressing sh-circ_0001361 or sh-NC were inoculated into the nude mice. The results indicated that the tumor volume and weight were obviously decreased by down-regulation of circ_0001361 compared with that in sh-NC group (Fig. 8A–C). In addition, the levels of circ_0001361, miR-525-5p, and VMA21 in tumor tissues were determined. The data displayed that circ_0001361 level was down-regulated, but miR-525-5p level was enhanced in sh-circ_0001361 group (Fig. 8D and E). Besides, the mRNA and protein levels of VMA21 in tumor tissues were evidently reduced by silencing of circ_0001361 (Fig. 8F and G). Collectively, depletion of circ_0001361 suppressed xenograft tumor growth via regulating miR-525-5p/VMA21 axis.

## Discussion

LUAD has been recognized as a primary contributor to tumor-related deaths worldwide[23]. Due to its high mortality and poor prognosis[24, 25], it is necessary to identify novel effective treatment for LUAD. The aberrant expression of circRNAs has been documented to affect the development of LUAD, suggesting circRNAs as potential therapeutic targets for LUAD. Circ_0001361 was reported to function as an oncogene to drive bladder cancer metastasis[17], which aroused our great interest in elucidating its role in LUAD. In this study, up-regulation of circ_0001361 was verified in LUAD tissues and cells. Inhibition of circ_0001361 restrained the proliferation, migration and invasion of LUAD cells. Further experimental data indicated that circ_0001361 could act as a sponge of miR-525-5p to increase VMA21 expression, which facilitated the growth and metastasis of LUAD cells. Therefore, our findings firstly identified the oncogenic roles and mechanisms of circ_0001361 in LUAD.

Due to the stable circular structure, circRNAs have been widely applicated as biomarkers for LUAD. For example, circ_0013958 expression was positively correlated with lymphatic metastasis of LUAD patients [26]; a negative association between circCRIM1 and lymphatic metastasis and TNM stage of LUAD was verified [27]. Additionally, increasing evidence has demonstrated the functional roles of circRNAs in the progression of LUAD [13, 14, 28]. circXPO1 was responsible for LUAD progression and might be a therapeutic target for this disease [29]. Despite all this, the regulatory mechanisms of circRNAs in LUAD remain far from being completely elucidated. In the present study, the aberrant up-regulation of circ_0001361 was found in LUAD for the first time. In vitro and in vivo experiments demonstrated that circ_0001361 contributed to the tumorigenesis of LUAD. Our observations firstly reported the tumorigenic effect of circ_0001361 in LUAD.

Recent studies have suggested that circRNAs can function as miRNA sponge to affect the expression of downstream target genes in different cancer types. For instance, circRNA-AKT1 contributed to cervical cancer progression via sequestering miR-942-5p to enhance AKT1 expression [30]. In triple-negative breast cancer, circGNB1 promoted growth and metastasis via sponging miR-141-5p to upregulate IGF1R [31]. It has been also suggested that circRNAs, mainly located in cytoplasm, can act to sequester miRNAs [9]. In this study, we disclosed that circ_0001361 mainly distributed in cytoplasm of LUAD cells and served as a sponge for miR-525-5p. The dysregulation of miRNAs participates in the occurrence and development of various tumors [32]. As reported by a previous literature, miR-525-5p was verified to be a tumor suppressor in cervical cancer via hampering epithelial-to-mesenchymal transition and anoikis resistance through UBE2C/ZEB1/2 signaling pathway [33]. So far, however, the biological function of miR-525-5p in LUAD has not been revealed. In this study, miR-525-5p was identified to be down-regulated in LUAD tissues and cells. Inhibition of circ_0001361 resulted in increased expression of miR-525-5p in LUAD cells. Rescue experiments further demonstrated that overexpression of miR-525-5p reversed circ_0001361-mediated proliferation and metastasis of LUAD cells. Therefore, these data suggested that circ_0001361 could sponge miR-525-5p to facilitate LUAD tumorigenesis.

VMA21 is a factor for lysosomal vacuolar ATPase assembly, and pathogenic deficiency of VMA21 has been verified to cause autophagic vacuolar myopathy [34] and autophagic liver disease [35]. VMA21 has been documented to be negatively regulated by miR-18b-5p, which was involved in the progression of LUAD [21] and cervical cancer [36]. In the present study, VMA21 was confirmed as a downstream target of miR-525-5p. Overexpression of circ_0001361 or inhibition of miR-525-5p promoted VMA21 expression in LUAD cells. Additionally, miR-525-5p-mediated anti-tumor effects were counteracted by VMA21 overexpression in LUAD cells. Our findings revealed that miR-525-5p/VMA21 axis was implicated in circ_0001361-mediated tumorigenic function in LUAD.

## Conclusion

Taken together, our results indicated that circ_0001361 expression was increased, while miR-525-5p level was reduced in LUAD tissues and cells. Furthermore, circ_0001361 silence suppressed the proliferation, migration, and invasion of LUAD cells in vitro and in vivo. Mechanistically, circ_0001361 sponged miR-525-5p to enhance VMA21 expression. Our study uncovers the roles and mechanisms of the novel circ_0001361 in LUAD cells, and provides theoretical foundation for circ_0001361 as a potential biomarker of prognosis and a therapeutic target in the clinical setting against LUAD. However, further studies need to be carried out to evaluate the potential application in clinical practice.

## Supplementary Information

**Additional file 1**: **Figure S1**. Supplementary data. (A) The putative binding sites between miR-525-5p and circ_0001361. (B) The predicted miR-525-5p binding sites in VMA21 3’UTR. (C) RT-qPCR was used to detect the levels of miR-525-5p and miR-520a-5p in A549 and PC9 cells. (D) The expression of VMA21 in LUAD samples and normal samples was evaluated by StarBase database. (E) The correlation between VMA21 transcript and circ_0001361 in 32 LUAD samples was determined by Pearson correlation analysis.

## Data Availability

All data generated or analysed during this study are included in this published article.

## Abbreviations

LUAD: Lung adenocarcinoma
circRNAs: Circular RNAs
VMA21: Vacuolar ATPase assembly factor VMA21
sh-circ_0001361: SiRNA targeted circ_0001361
sh-NC: SiRNA negative control
mimic NC: Mimics negative control
GAPDH: Glyceraldehyde 3‐phosphate dehydrogenase
DMSO: Dimethylsulfoxide
HBE: Human bronchial epithelial
WT: Wild type
MUT: Mutant
RIP: RNA immunoprecipitation
bio-miR-525-5p-WT: Biotinylated WT miR-525-5p
bio-miR-525-5p-MUT: Mutant miR-525-5p
SD: Standard deviation
