# Correction to: Hsa_circ_0001361 facilitates the progress of lung adenocarcinoma cells via targeting miR-525-5p/VMA21 axis

**DOI:** 10.1186/s12967-021-03140-6

**Published:** 2021-11-25

**Authors:** Hong-Yu Shen, Liu-Xi Shi, Lin Wang, Le-Ping Fang, Wei Xu, Ju-Qing Xu, Bo-Qiang Fan, Wei-Fei Fan

**Affiliations:** 1 Department of Hematology and Oncology, Department of Geriatric Lung Cancer Laboratory, Geriatric Hospital of Nanjing Medical University, Jiangsu Province Geriatric Hospital, No.65 Jiangsu Road, Gulou District, Nanjing, 210000 Jiangsu People’s Republic of China; 2 GCP Office, The Second Affiliated Hospital of Soochow University, Suzhou, 215000 Jiangsu People’s Republic of China; 3 Department of Oncology, The First Affiliated Hospital of Nanjing Medical University, Nanjing, 210000 Jiangsu People’s Republic of China

## Correction to: J Transl Med (2021) 19:389 https://doi.org/10.1186/s12967-021-03045-4

Following publication of the original article [1], the authors identified an error in the order of e-mail addresses.

The original publication lists the contacts as followed:

*Correspondence: bq_fan@139.com; fwfei1974@njmu.edu.cn.

However, the correct order of contacts is:

*Correspondence: fwfei1974@njmu.edu.cn; bq_fan@139.com.

This is due to Dr. Fan being the main corresponding author.

The original article has been updated to reflect the correct order.
