# Supplementary material for: Hsa_circ_0001361 facilitates the progress of lung adenocarcinoma cells via targeting miR-525-5p/VMA21 axis
Source: J Transl Med. 2021 Sep 10;19:389. doi: 10.1186/s12967-021-03045-4 (PMC8434718; doi:10.1186/s12967-021-03045-4)
Supplement: Supplementary file 1 — Additional file 1: Figure S1. Supplementary data. (A) The putative binding sites between miR-525-5p and circ_0001361. (B) The predicted miR-525-5p binding sites in VMA21 3’UTR. (C) RT-qPCR was used to detect the levels of miR-525-5p and miR-520a-5p in A549 and PC9 cells. (D) The expression of VMA21 in LUAD samples and normal samples was evaluated by StarBase database. (E) The correlation between VMA21 transcript and circ_0001361 in 32 LUAD samples was determined by Pearson correlation analysis. [file 12967_2021_3045_MOESM1_ESM.pdf]

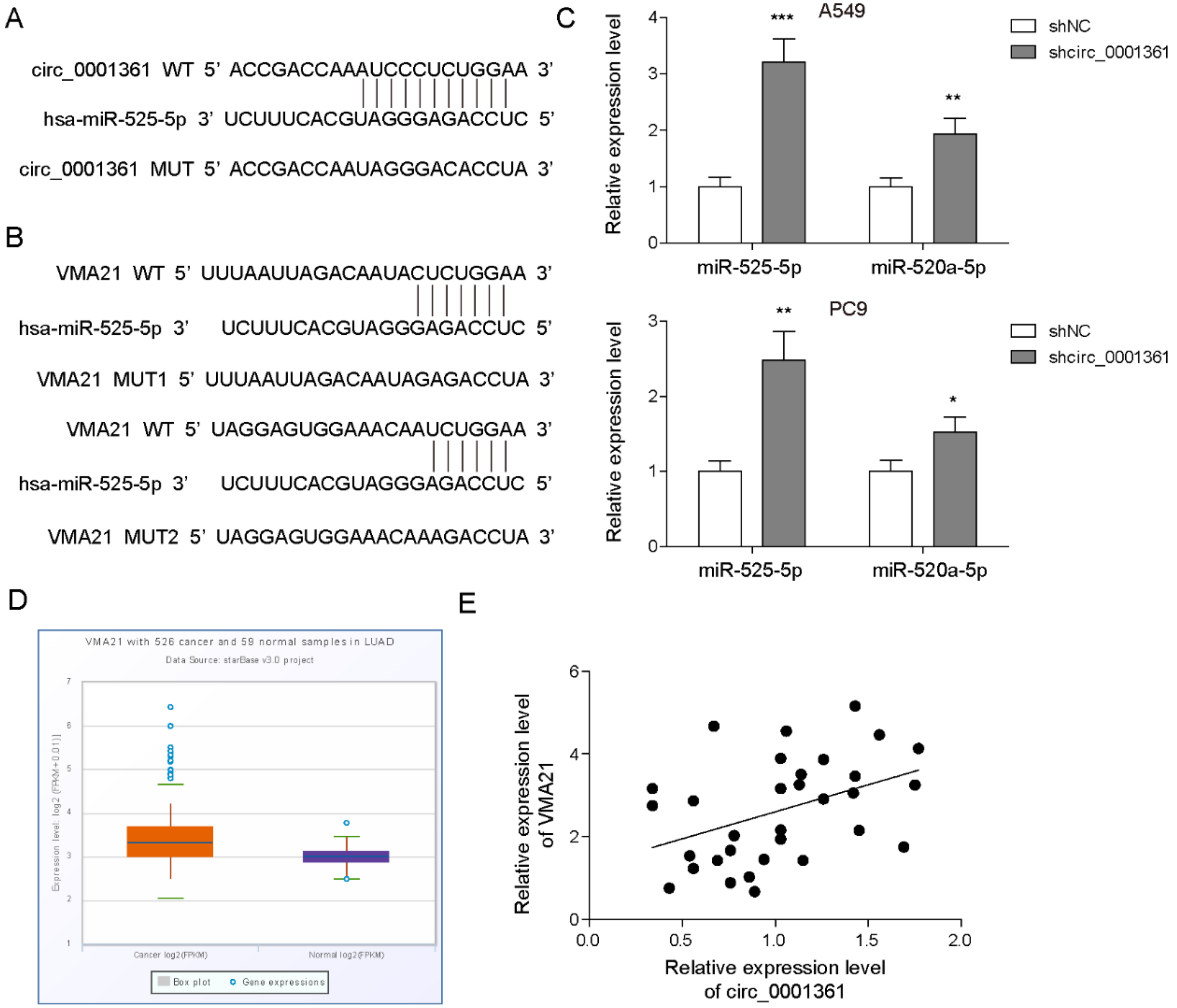

Supplementary Figure legend. (A) The putative binding sites between miR-525-5p and circ_0001361. (B) The predicted miR-525-5p binding sites in VMA21 3'UTR. (C) RT-qPCR was used to detect the levels of miR-525-5p and miR-520a-5p in A549 and PC9 cells. (D) The expression of VMA21 in LUAD samples and normal samples was evaluated by StarBase database. (E) The correlation between circ_0001361 and VMA21 transcript in 32 LUAD samples was determined by Pearson correlation analysis.
